# Morphological changes after radiosurgery for mesial temporal lobe epilepsy

**DOI:** 10.1007/s00701-015-2525-2

**Published:** 2015-08-16

**Authors:** Zdeněk Vojtěch, Hana Malíková, Martin Syrůček, Lenka Krámská, Jan Šroubek, Vilibald Vladyka, Roman Liščák

**Affiliations:** Department of Neurology, Na Homolce Hospital, Roentgenova 2, 15030 Prague 5-Motol, Czech Republic; Department of Radiodiagnostics, Na Homolce Hospital, Prague, Czech Republic; Department of Pathology, Na Homolce Hospital, Prague, Czech Republic; Department of Psychology, Na Homolce Hospital, Prague, Czech Republic; Department of Neurosurgery, Na Homolce Hospital, Prague, Czech Republic; Department of Stereotactic and Radiation Neurosurgery, Na Homolce Hospital, Prague, Czech Republic

**Keywords:** Gamma knife, Mesial temporal sclerosis, Epilepsy surgery, Radionecrosis

## Abstract

**Background:**

To review our experience with morphological developments during the long-term follow-up of patients treated by Gamma Knife radiosurgery for mesial temporal lobe epilepsy.

**Method:**

Between 1995 and 1999, we treated 14 patients with marginal doses of 24 Gy (*n* = 6) and 18–20 Gy (*n* = 8). Nine of these were operated on for insufficient seizure control. We reviewed seizure outcome and magnetic resonance images in both operated and unoperated patients and also re-examined histopathology specimens.

**Results:**

Of the nine operated patients, two were Engel IIIA, one was IVA, five were IVB, and one was Engel IVC prior to surgery. At their final visit, five cases had become Engel class IA, one patient was ID, and two were IIC. In one patient the follow-up was not long enough for classification. Of the five unoperated patients, one was Engel class IB, one was IIIA, one IIB and one IVB at their final visit. Radionecrosis developed in 11 patients, occurring more often and earlier in those treated with higher doses. Collateral edema reached outside the temporal lobe in six patients, caused uncal herniation in two and intracranial hypertension in three. During longer follow-up, postnecrotic pseudocysts developed in 9 patients, and postcontrast enhancement persisted for 2.5–16 years after GKRS in all 14 patients. In five of them we detected its progression between 2 and 16 years after treatment. Signs of neoangiogenesis were found in two patients and microbleeds could be seen in five. Histopathology revealed blood vessel proliferation and macrophage infiltration.

**Conclusions:**

Early delayed complications and morphological signs suggesting a risk of development of late delayed complications are frequent after radiosurgery for mesial temporal lobe epilepsy. Together with its unproven antiseizure efficacy, these issues should be taken into account when planning future studies of this method.

**Electronic supplementary material:**

The online version of this article (doi:10.1007/s00701-015-2525-2) contains supplementary material, which is available to authorized users.

## Introduction

Mesial temporal lobe epilepsy (MTLE) with hippocampal sclerosis (HS) is the most common epilepsy syndrome in adults and is often associated with pharmacoresistance [31]. In these cases, epilepsy surgery is an effective treatment option, and patients have a 60–80 % chance of a seizure-free outcome [8]. However, procedure-related complications are not negligible [14].

The need to minimize complication rates and potentially to improve neuropsychological outcomes provides an impetus for the development of innovative treatments. Gamma Knife radiosurgery (GKRS) for MTLE is an extremely attractive treatment modality.

There are three lines of evidence that GKRS might work in MTLE:

1. Experiments on animals with limbic epilepsy produced significant reductions in both the frequency and duration of seizures [5]. Animal and human data suggest that GKRS may have anticonvulsive effects in subnecrotic doses [38]. Proposed mechanisms include neuromodulatory effects on different enzymes and secondly on amino acids and the selective modification of the function of epileptic neurons [26]. However, a relationship was found in animal models between increasing the radiosurgical dose and the decreasing frequency of seizures [23]. On the other hand, doses higher than 50 Gy caused edema and necrosis [19].
2. The antiepileptic effectiveness of GKRS in patients with symptomatic epilepsies due to arteriovenous malformations (AVM), brain tumors, cavernomas and hypothalamic hamartomas [22]. In epilepsies associated with AVM, improved control of seizures is significantly more likely with complete AVM obliteration (82 % and 41 % seizure-free, respectively) [20]. Studies on tumors indicate that temporal localization and larger doses of radiation outside the lesion border are associated with a favorable outcome [28].
3. Attempts at radiosurgical interventions indicated for primarily epileptological reasons performed by interstitial brachytherapy [34], radiotherapy by cobalt irradiator [2] and fractionated radiotherapy [33].

In 1995, Régis et al. reported preliminary results from the 16-month follow-up of the first patient treated for MTLE/HS [25]. He had been seizure free since the procedure, and neuroimaging showed selective injury to the amygdaloentorhinohippocampal target.

Since then three things have become apparent:

1. Seizure control rates reported from different centers vary. Favorable seizure outcome comparable to open surgery has been reported by some authors with 65–67 % of seizure-free patients [3]. In patients treated with higher doses, the proportion of seizure-free patients reported was even higher (76.9 %) [1]. The seizure remission rate was lower in other studies (0–37.5 %) [32, 37, 18, 6], some of which used similar treatment parameters to more successful studies, so the reason for these discrepancies is not clear. The rate of neurological complications (e.g., visual field defects) was probably comparable to open surgery [15], while the benefit in the neuropsychological domain was questionable [21].
2. Although GKRS is generally considered a safe treatment modality, complications after treatment are documented [10]. They can be classified as early and late. Early complications occur months after treatment and include radiation-induced edema, which may be associated with seizure worsening and focal neurological deficits (e.g., verbal memory decline) [11]. In approximately 15 % of patients, edema causes signs of intracranial hypertension and requires corticosteroid therapy. Delayed seizure control might pose a heightened risk of sudden unexplained death. Late complications appear years after treatment and consist of delayed radionecrosis and cyst formation. Worsening and de novo development of seizures, intractable epilepsy and HS have been described [16]. Furthermore, seizure relapse was reported in all seizure-free patients during attempts at drug reduction [1].
3. GKRS has become a treatment option in focal lesions that are otherwise difficult to access through open surgery (e.g., hypothalamic hamartomas) [12]. However, seizure freedom was lower (~30 %) compared to open surgery (~50 %). In the treatment of MTLE, GKRS has not gained widespread acceptance [29].

The aim of this study is to review our experience with development of morphology during long-term follow-up of patients treated by GKRS for MTLE.

## Materials and methods

### Patients

Between November 1995 and May 1999, we diagnosed 14 consecutive patients with MTLE/HS and treated them with GKRS (Supplementary material, Table 1). We described our group’s characteristics, the presurgical evaluation protocol and seizure outcome in our previous article [37]. To briefly recapitulate, there were eight female and six male patients in whom epilepsy began at a mean age of 10.9 years (2.5–38 years) and whose mean age at the time of treatment was 33.4 years. By the time GKRS was performed, the patients had had epilepsy for an average of 23.2 years (9–46). Early risk factors were present in 12 patients. These were mostly febrile seizures; one patient (patient 1) had a history of purulent meningitis, and the other (patient 6) had experienced repeated afebrile generalized seizures since an early age. All the patients had complex partial seizures (CPSs), and auras appeared either independently or at the beginning of the seizure in six of them. CPS occasionally led to secondary generalization in four patients.

Each patient underwent a routine presurgical examination [neurologic and neuropsychological testing, repeated interictal electroencephalography (EEG), scalp video-EEG study with ictal recording, magnetic resonance imaging (MRI) and intracarotid Amytal test].

In addition to a standard scalp 10–20-electrode system, sphenoidal electrodes were used for the interictal video-EEG evaluation in two patients (patients 1, 2). Foramen ovale electrodes for ictal recording were inserted in seven patients (patients 2, 4–9). In one patient (patient 9) noninvasive data were inconclusive, and an invasive study was performed with a combination of strip and depth electrodes. A similar electrode combination was also used in patient 1, as temporal lobe atrophy found on MRI did not exclude neocortical temporal lobe epilepsy.

The MRI study included a T1-weighted three-dimensional acquisition, a tilted coronary T2-weighted acquisition with a long second echo, and fluid-attenuated inversion-recovery sequences. Unilateral MTLE was diagnosed in all patients.

The patients underwent GKRS targeted to the medial temporal lobe (to the amygdala, sparing the superior and mesial part, to the head and anterior half of the body of hippocampus, and to the anterior part of the parahippocampal gyrus). The radiosurgical parameters for six of our patients (patients 2, 4–8) were the same as those described by Rėgis et al. [25]. Prominent radiation-induced responses led us to reduce the dose and volume in patients 3 and 9–14. The mean irradiated volume for the whole group was 6.764 mm^3^. In patients treated with 18–20 Gy, it was 6.388 mm^3^ on average (ranging from 5.200 to 8.900), whereas in those irradiated with 25 Gy, the average volume was 7.267 mm3 (6.600–7.700).

We offered all our patients the option of open surgery, and five of them refused. In the remaining nine patients, an anterior temporal lobectomy was performed on the side treated by radiosurgery because of insufficient seizure control. No surgery was indicated for any of the patients for either early or late complications.

Mean follow-up was 191.5 months (median 190.5 months, range 175–217). Operated patients were monitored for a mean 98.4 months (median 83, range 61–198) before the operation and 93.2 months (median 102, range 12–153) after the operation. Unoperated patients were followed up for a mean 191.2 months (median 181, range 175–217).

### Methods

A certified epileptologist reviewed seizure outcome and classified both operated and unoperated patients according to Engel’s classification after GKRS, before the operation and at the last check-up. As the effect of GKRS is delayed and the patients’ seizure status may change over time, we modified the original Engel classification and presented outcome figures are calculated from seizure outcomes 2 years prior the visit. A certified neuroradiologist reviewed follow-up MRI scans and searched for signs of delayed complications. An experienced neuropathologist re-examined the available histological material from operated patients. This study was approved by the hospital Ethics Committee.

### MRI

During the first 2 years after GKRS, patients followed a standard 3-monthly schedule for MRI follow-up controls. MRI evaluations were performed on a 1.5-T whole-body MR system (Magnetom, Siemens) according to the following protocol: TSE T2/PD WI axial, T2 WI turbo FLAIR axial, TSE T2 WI coronal, SE T1WI coronal plane, and SE T1WI coronal and axial planes after application of gadolinium intravenous contrast agents. Subsequently, for up to 4 years after GKRS, MRI follow-ups were indicated according to the patients’ clinical status, but at least once a year. Patients indicated for re-operation by anteromesial temporal lobectomy (AMTL) underwent a preoperative three-dimensional volume acquisition sequence (FLASH 3d) after administration of intravenous gadolinium contrast agents. Other MRI evaluations were inconstant and were dependent on the patients’ clinical status. All patients were scheduled for late MRI follow-up 16–18 years after GKRS. The late MRI follow-ups were performed on a 3-T whole-body MRI system (Skyra, Siemens) following the standard protocol in common diagnostic sequences: T2 3D, T2 WI turbo FLAIR axial and coronal, TSE T2 WI coronal (coronal planes were orientated perpendicular to the long axis of the hippocampus), SE T1 WI sagittal, SWI 3D axial, DWI axial and SE T1WI coronal and axial planes after administration of gadolinium intravenous contrast agents.

### Histological evaluation

In all cases material was examined by microscope in sections from formaldehyde-fixed and paraffin-embedded blocks. Sections (4 μm thick) were stained using routine methods (hematoxyline-eosin, periodic acid-Schiff (PAS), Masson’s trichrome). Selected slides were examined by imunohistochemistry using monoclonal antibodies—map2c (DAKO), CD 68 (DAKO), p53 (DAKO), S100protein (DAKO), GFAP (DAKO) and KI67 (DAKO).

## Results

### Seizure outcome

At the time our first patients underwent AMTL (39 months), 1 patient was Engel IIA, seven were IIIA, five were IVB and one Engel IVC. Of nine operated patients, two were Engel IIIA, one was IVA, five were IVB and one Engel IVC before the operation. At their last visit, they had become Engel class IA in five cases, one patient was ID and two IIC. In one patient (patient 2) the follow-up was not sufficiently long to justify classification. Of the five unoperated patients, one was Engel class IB, one IIIA, one IIB and one IVB at their last visit. There was one death unrelated to epilepsy as a seizure-free patient (patient 4) died from influenza 127 months after AMTL.

### MRI results

**Fig. 1:**
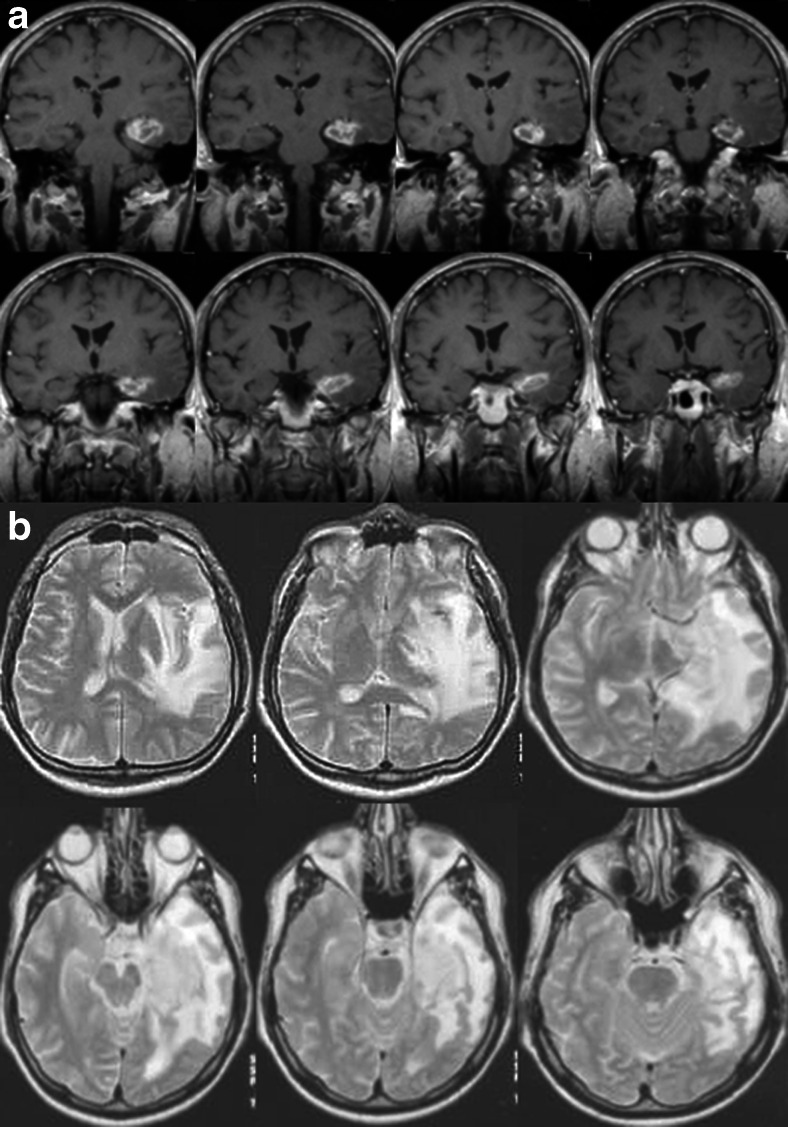
**a** Peripheral ring enhancement 2 months after GKRS in patient 2. **b** Large perifocal edema reaching outside the temporal lobe and midline shift developed 11 months after GKRS in the same patient

In the group as a whole, our short-term evaluation found edema identified as a T2-weighted hyperintensity around a T1 contrast-enhancing volume in 11 patients. Collateral edema reached outside the temporal lobe in six patients (Fig. 1). It caused uncal herniation in two patients and intracranial hypertension in three. We classifed these findings as radionecrosis. This classification is supported by the fact that in 9 of these 11 patients postnecrotic pseudocysts developed during follow-up.

**Fig. 2:**
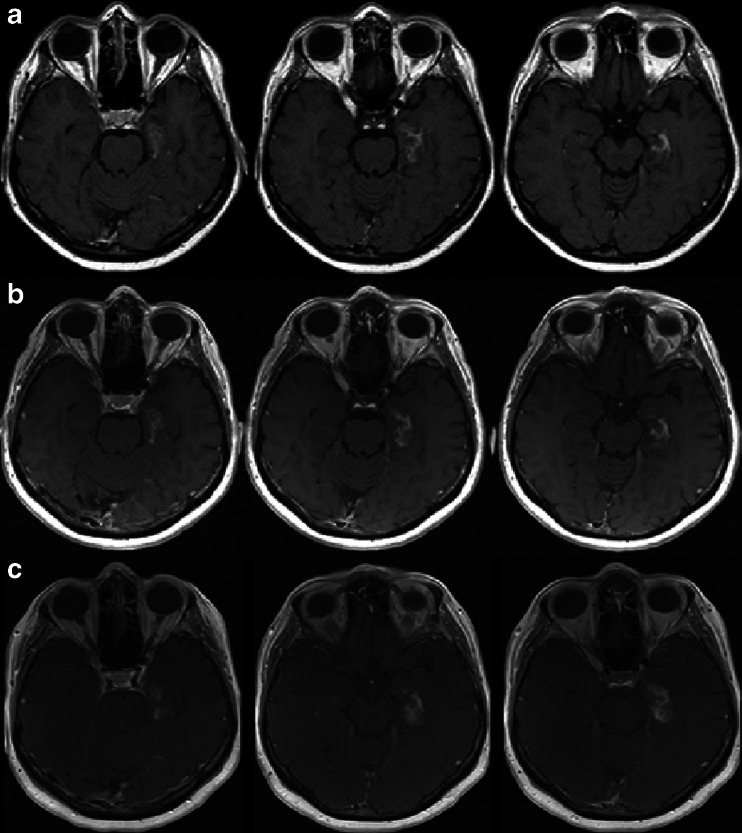
Postgadolinium contrast enhancement (SE T1 transversal) of postradiation changes in the medial part of the left temporal lobe; progression through years (**a** row 7 years, **b** row 10 years, **c** row 15 years after therapy) found in patient 14

**Fig. 3:**
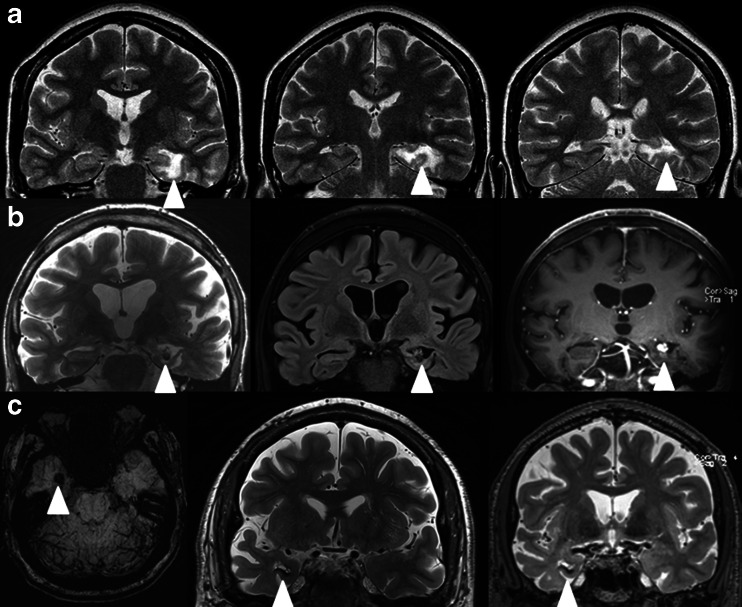
**a** White matter changes after radiosurgery of the left mesial temporal structures 15 years after therapy (TSE T2 coronal scans perpendicular to the long axis of the hippocampus) in patient 13 (*arrowheads*). **b** The angioproliferative nodule 15 years after treatment in patient 11 (*right picture*, TSE T2 coronal; *middle picture*, FLAIR coronal; *left picture*, SE T1 after gadolinium contrast intravenous administration coronal scan) (*arrowheads*). **c** Microbleed 16 years after GKRS in patient 11 (*arrowheads*)

On longer term follow-up, postcontrast enhancement persisted for 2.5–16 years after GKRS in all 14 patients, and in 5 of these we detected its progression between 2 and 16 years after treatment (Fig. 2). In six patients we noted permanent collateral damage outside the target structures with gliosis of the white matter, in two patients signs of neoangiogenesis were found, and in five microbleeds could be seen (Fig. 3). Postoperative ischemia developed in four patients in areas where edema had been identified earlier. In two patients an optic tract lesion was present.

We investigated whether radiation doses and volumes treated influenced either the early or late complication rate. For this purpose we divided patients into lower (18–20 Gy) and higher (25 Gy) dose groups (Table 1). As mentioned above, lower doses were targeted to somewhat smaller volumes.

**Table 1:** Early and late MRI changes in treated patients

Case	Early delayed changes	Late delayed changes
1	RE (18 m; 40 × 30 × 30 mm), EOTL	PNPC, GOTS, PCE (2.5 years)
2	RE (11 m; 40 × 30 × 30 mm), EOTL, UH, MS,	PNPC, GOTS, PCE (8 years), PPCE (8–16 years), MB, NAG, POI (TO, Th)- HA
3	RE (12 m, 40 × 25 × 2 5 mm), EOTL, MS	PNPC, PCE (6 years), POI
4	RE (11 m; 32 × 22 × 20 mm)	PNPC, PCE (3 years)
5	RE (12 m; 55 × 30 × 36 mm), EOTS (BG, TO), MS	PNPC, GOTS, PCE (17 years), MB
6	RE (14 m; 43 × 37 × 27 mm), EOTL, UH	PNPC, PCE (3 years), PPCE (3–12 years), GOTS (TO), MB, OTL (16 m), QA
7	RE (9 m; 31 × 26 × 20 mm), EOTL (CE, CI, BG, Th)	PNPC, PCE (8 years), OTL, POI (CI, Th)
8	RE (18 m; 20 × 10 × 10 mm), TLE	PNPC, PCE (2 years), PPCE (2–3.5 years)
9	ETS, TLE	PCE (16 years), POI
10	RE (24 m; 30 × 18 × 10 mm), TLE	PNPC, PCE (16 years), MB
11	RE (36 m; 12 × 11 × 18 mm), TLE	PNPC, GOTS, PCE (16 years), MB, NAG (16 years)
12	ETS	PCE (4 years)
13	ETS (8 × 7 × 5 mm)	GOTS, PCE (5 years), PPCE (5–8 years)
14	RE (18 m; 20 × 25 × 15 mm), TLE	PCE (4 years), PPCE (4–15 years), MB

Lower- and higher-dose subgroup results are highlighted with blue and red color, respectively

*Abbreviations*: *BG* basal ganglia, *CI* internal capsule, *EOTL* expansive edema outside the temporal lobe, *ETS* enhancement and edema of target structures, *GOTS* gliotic changes outside target structures, *HA* hemianopia, *MB* microbleeding, *MS* midline shift, *NAG* neoangiogenesis, *OTL* optic tract lesion, *PCE* persisting postcontrast enhancement, *PNPC* postnecrotic pseudocyst, *POI* postoperative ischemia, *PPCE* progression of enhancement (duration in years in brackets), *QA* quadrantanopia, *RE* ring enhancement, *SETS* small enhancement of target structures, *Th* thalamus, *TLE* temporal lobe edema, *TO* temporo-occipital region, *TP* temporal pole, *UH* uncal herniation

In all patients treated with 25 Gy to the 50 % isodose (*n* = 6), radionecrosis developed 9–14 months after GKRS. Enhancement was found to roughly correspond to the 50 % isodose line. Collateral edema was confined to the temporal lobe in two patients (patients 4 and 8). In the remaining four patients it reached outside the temporal lobe, both dorsally (occipital lobe) and medially (basal ganglia, internal capsule, thalamus). In two patients it caused midline shift (patients 2 and 5) and in two cases uncal herniation (patients 2 and 6). Clinical symptoms of intracranial hypertension appeared in patients 6 and 7 and required hospitalization and intravenous corticosteroid therapy.

During follow-up, postnecrotic pseudocysts developed in all six patients. Postcontrast enhancement also persisted for 3–9 years after GKRS in all patients. We found progression of postcontrast enhancement between 8 and 16 years after GKRS in patient 2, between 3 and 15 years in patient 6 and between 2 and 3.5 years in patient 8. In patient 4 postcontrast enhancement in the target structures persisted until open surgery, which was performed 39 months after GKRS. Prominent gliosis outside the target structures was present in patients 2, 5 and 6. Signs of optic tract degeneration were evident in patients 6 and 7. Notably, in both these patients intracranial hypertension should be treated by intravenous corticosteroids. In patient 6 edema gradually developed into the optic tract lesion, which was apparent from 16 months after GKRS and caused quadrant anopia. In patient 7 a small optic tract lesion was evident 8 years after GKRS, but no gross visual field defects were evident on neurological evaluation. Microbleeding was present in patients 2, 5 and 6. After a subsequent operation ischemia developed in patient 2 (in the temporo-occipital region and thalamus) and caused hemianopia. In patient 7 (internal capsule and thalamus) it was clinically silent. Notably, both these complications were found in patients in whom collateral edema had reached outside the temporal lobe. It seems that edema in the early delayed phase makes the areas where it occurs more vulnerable to ischemia later.

The degree of radiologic changes in the lower-dose subgroup (*n* = 8) was somewhat more variable. Formation of radionecrosis was delayed by 12–24 months and developed in both patients treated with 20 Gy. In patient 1 it reached outside the target structures to the temporopolar region. However, radionecrosis was found to develop even in patients treated by 18 Gy (patients 10 and 11). Collateral edema reaching outside the temporal lobe only formed in those treated with 20 Gy (patients 1 and 3), and in one of them (patient 3) it caused a midline shift with signs of intracranial hypertension requiring oral corticosteroids. Interestingly, this patient was also treated by insulin for diabetes. Diabetes is identified as a possible predisposing factor for the development of radionecrosis [24]. In another patient (patient 1) large radionecrosis and collateral edema did not cause clinically manifest intracranial hypertension, presumably because of preexisting ipsilateral temporal lobe atrophy. In the remaining patients in whom radionecrosis did not develop (patients 9, 11, 12, 13), the most frequent finding was small edema and enhancement of target structures, which occurred 11–36 months after GKRS.

Postcontrast enhancement persisted for up to 4–7 years. Progression of this enhancement was noted in patients 9, 10 and 14 beginning 5 years after GKRS and lasting until surgery and between 3–16 years and 4–15 years after GKRS, respectively. Gliosis outside the target structures was noted in patients 1, 9, 11 and 14. Signs of microbleeding were present in patients 11 and 14. Postoperative ischemia was found in patient 3.

### Histopathological evaluation

**Fig. 4:**
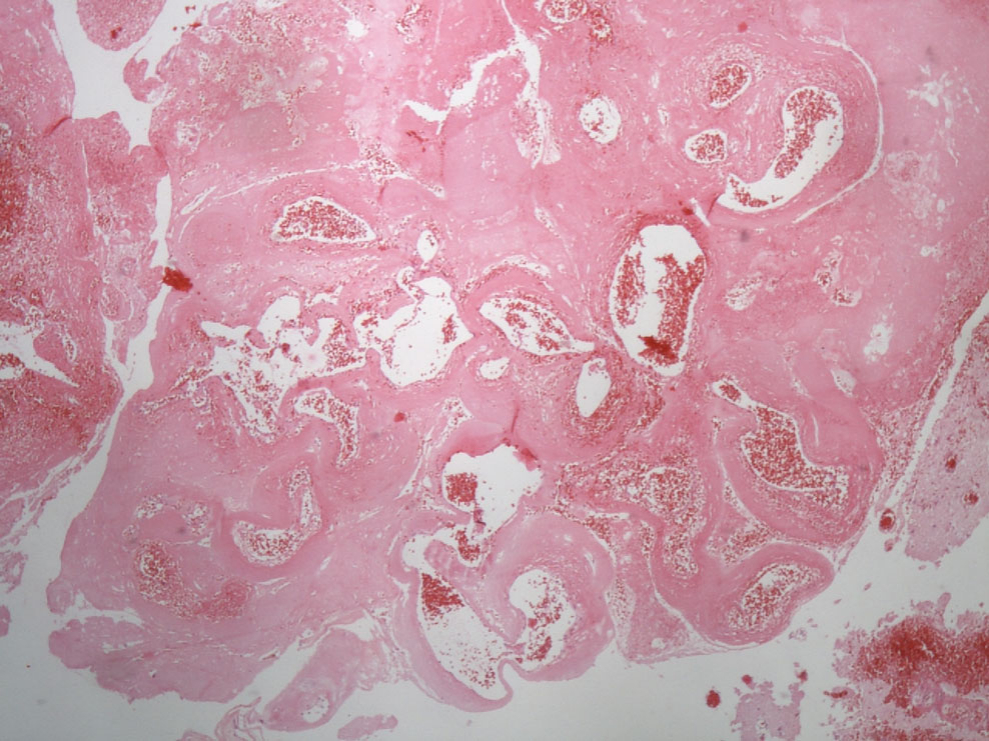
Angiomatous proliferation with partially obstructed and recanalized blood vessels 16 years after GKRS in patient 2 (hematoxilin-eosin, magnification 40×)

**Fig. 5:**
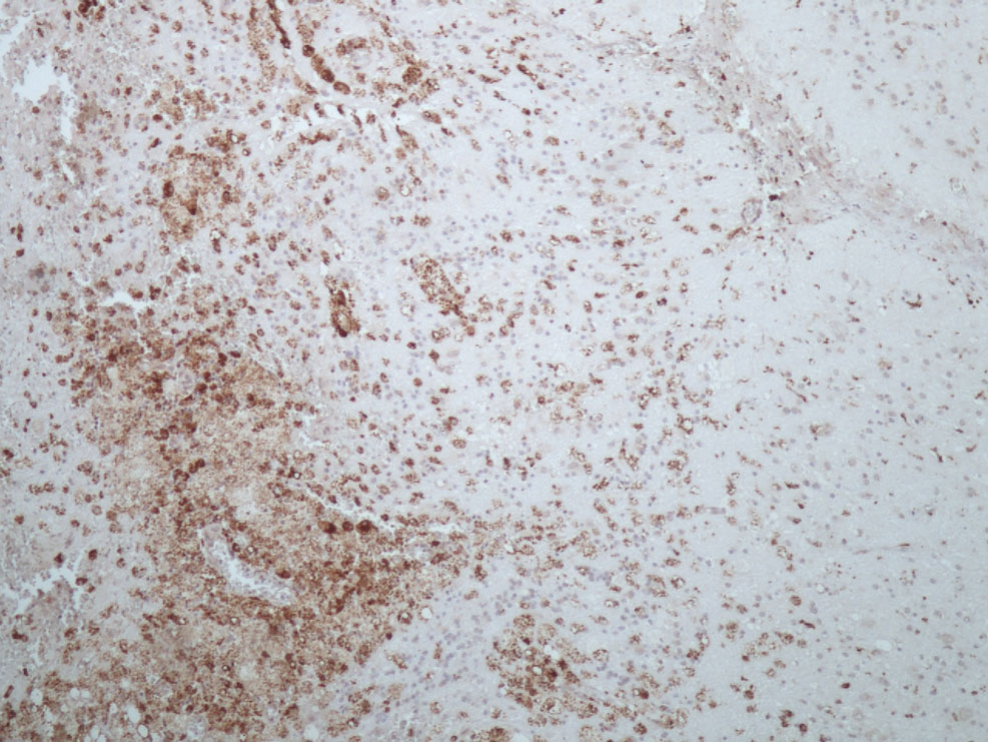
Perivascular macrophage infiltration (patient 3) 6 years after GKRS (CD 68, magnification 100×)

Unfortunately, histology specimens were only available for revision in seven patients (2, 3, 6, 7, 9, 12, 13). Microscopically, we found late postradiosurgery effects in all cases. In patients 2, 9 and 12 thickened walls of blood vessels with obliterated lumens and necrosis were observed in the parahippocampal gyrus and hippocampus. Proliferation of the blood vessels and evidence of recanalization mimicked AVM (Fig. 4). In patient 6 fibrinoid necrosis, gliosis and slight angiogenesis were observed. In patients 3, 7 and 13 only gliotic changes were found. The surrounding gliotic tissue can be focally calcified. In other biopsy specimens we found reactive gliosis with pseudocysts of various sizes and with macrophage infiltration (Fig. 5). Similar changes along with perioperative damage were described in both discarded specimens.

## Discussion

The use of GKRS for intractable MTLE began in the mid 1990s. Various seizure outcomes have been reported in the literature since that time. In our group of 14 patients none became entirely seizure-free after the treatment. One patient from the lower-dose subgroup was Engel IB at her last visit. Of the patients who underwent subsequent operations, a seizure-free outcome was achieved in five of eight patients.

Marginal doses of 20–25 Gy to the 50 % isodose were reported to be effective for seizure control. These doses usually cause destructive reactions in the targeted structures and collateral edema [17]. These well-known complications could cause transient seizure worsening, intracranial hypertension and a delayed effect of GKRS. These alterations occurred approximately 9–12 months after the treatment and gradually changed into nonhomogeneous atrophy in the target area by the end of the second year. They are thought to be required for successful seizure control [4].

Radiation injury is related to numerous factors, of which dose and volume are paramount [18]. However, the degree of radiographic changes is extremely variable and unpredictable [29]. It is influenced by the type of tissue treated and location of the lesion. Individual sensitivities to radiation injury may be affected by genetic factors (e.g., DNA repair mechanisms) or even acquired conditions, such as intracranial atherosclerosis. It is widely accepted that normal brain tissue (as is the case in most cases of HS) is more vulnerable to radiation injury than brain tumors [13].

In our group of patients, radionecrosis and collateral edema were more likely to occur and developed earlier in those treated with higher doses. In a lower dose subgroup various degrees of tissue destruction could be seen on MRI in patients treated with similar protocols. Intracranial hypertension may develop in vulnerable patients treated with lower doses. We reported on the functional consequences (worsening seizure frequency, headaches) in our previous work [37]. Some authors advocate the view that collateral damage is reversible and does not affect seizure control later on [27]. These changes were thought to constitute a fair trade-off in terms of the greater comfort for the patients and the minimally invasive nature of GKRS. In our material, however, we found extensive collateral damage (temporal lobe white matter, optic tract). These morphological alterations occur in patients treated by both lower and higher doses and can have functional consequencies (e.g., visual field defects).

Delayed radiation necrosis and cyst formation (incidence 2–30 %) were described after GKRS for various conditions. They can occur many years after treatment. Finet et al. were the first to report the case of a patient treated with GKRS for MTLE who developed intracranial hypertension because of a hemorrhagic lesion in the treated area with temporal conus requiring urgent surgery 6 years after GKRS [9]. Ganz and Reda described a man who developed similar changes identified by MRI, refused surgery and became blind because of remote radionecrosis [13]. Vale et al. referred to the case of a patient with early edema requiring corticosteroid treatment in whom late seizure recurrence occurred because of the formation of an enhancing cystic lesion in the mesial temporal lobe [36]. Usami et al. decribed a series of seven patients treated by 18–25 Gy to the 50 % isodose [35]. High-dose treatment was effective in abolishing seizures in two of five patients and significantly reduced seizure frequency in two others, but required open surgery because of symptomatic radionecrosis 5–10 years after treatment in two patients. In one patient who was not operated on, MRI showed radionecrosis 10 years after treatment. Kawamura et al. reported 11 patients, only 1 of whom was seizure-free and without symptomatic complications during long-term follow-up [18]. However, even in this patient, extensive radiation-induced changes could be seen on MRI. Chen et al. reported on four patients (2 of them with MTLE), one of whom was Engel class IA, two of whom were unchanged (IVA) and one with worsened seizures (IVC) [6]. In all patients subsequent resection was performed for radionecrosis, which developed 7–12 years after the radiosurgical treatment. All the patients were Engel class I (3 patients I A and 1 patient I B) after the operation. Histologic examination in operated cases revealed lesions of the gray and white matter consisting of necrosis, neuronal loss and astrogliosis, and vascular changes with hyalinization, neovascularization, hemorrhagic foci and hemosiderin depositions.

The most common theory attributes the development of late radionecrosis and cyst formation to endothelial cell damage with progressive dysfunction of the blood-brain barrier [7]. Fibrinoid necrosis of blood vessel walls occurs first, followed by vessel wall thickening, hyalinization and telangiectasia. An influx of leukocytes leads to the overproduction of various cytokines, inducing oligodendrocyte apoptosis. Leakage of serum components into the brain tissue causes glial injury and fibrinoid coagulative degeneration with subsequent cyst formation. The development of delayed complications was suggested by the emergence of microbleeding, postcontrast enhancement and perifocal edema [30].

However, in our material we found some kinds of these signs, usually in combination, in every patient. Therefore, it follows that radiation-induced changes are still active many years after therapy. These morphological alterations may develop into more serious complications (late necrosis, degenerative angiomatosis) many years after treatment with significant negative functional, even life-threatening, consequences. Thus, any patient treated with GKRS for MTLE should be regularly screened for late complications. These facts should be taken into account when planning future studies of this method. This information could be of interest for physicians who refer their patients for this treatment.

Our study has many limitations: (1) The group of patients is small and inhomogeneous as treatment protocols vary. (2) There is no control group of patients treated by standard epilepsy surgery methods. However, it is hardly possible to have a control group for a historical case series. Historically, radiosurgery was used in a limited number of institutions. Therefore, long-term follow-up results are limited and thus valuable. (3) Histological specimens were not available for re-analysis in all patients. (4) It shows a single-center experience. (5) It is a retrospective study. However, its aim is not to give a comprehensive evidence-based account.

## Conclusion

The role of GKRS in the treatment of epilepsy is still unclear, although at present pharmacoresistant MTLE is a treatable condition. Microsurgery is a safe treatment that can achieve control of seizures immediately after surgery with the advantage that a histological diagnosis is obtained. After GKRS, there is a delay in antiseizure effects that carries a risk of mortality because of accidents and the possibility of sudden unexplained death in epilepsy. It seems that edema in the early delayed phase makes the areas where it occurs more vulnerable to ischemia during subsequent operations. GKRS for MTLE could cause serious late complications caused by vascular and inflamatory mechanisms. These changes may even occur in patients treated with lower doses. Our results stress the need for life-long MRI follow-up. These facts should be taken into account when planning future studies of this method.

## Electronic supplementary material

Supplementary material Table 1 (DOCX 14 kb)

## References

[1] Barbaro NM, Quigg M, Broshek DK, Ward MM, Lamborn KR, Laxer KD, Larson DA, Dillon W, Verhey L, Garcia P, Steiner L, Heck C, Kondziolka D, Beach R, Olivero W, Witt TC, Salanova V, Goodman R (2009). A multicenter, prospective pilot study of Gamma Knife radiosurgery for mesial temporal lobe epilepsy: seizure response, adverse events, and verbal memory. Ann Neurol.

[2] Barcia Salorio JL, Roldan P, Hernandez G, Lopez Gomez L (1985). Radiosurgical treatment of epilepsy. Appl Neurophysiol.

[3] Bartolomei F, Hayashi M, Tamura M, Rey M, Fischer C, Chauvel P, Régis J (2008). Long-term efficacy of Gamma Knife radiosurgery in mesial temporal lobe epilepsy. Neurology.

[4] Chang EF, Quigg M, Oh MC, Dillon WP, Ward MM, Laxer KD, Broshek DK, Barbaro NM, Epilepsy Radiosurgery Study Group (2010). Predictors of efficacy after stereotactic radiosurgery for medial temporal lobe epilepsy. Neurology.

[5] Chen ZF, Kamiryo T, Henson SL, Yamamoto H, Bertram EH, Schottler F, Patel F, Steiner L, Prasad D, Kassell NF, Shareghis S, Lee KS (2001). Anticonvulsant effects of gamma surgery in a model of chronic spontaneous limbic epilepsy in rats. J Neurosurg.

[6] Chen N, Du SQ, Yan N, Liu C, Zhang JG, Ge Y, Meng FG (2014). Delayed complications after Gamma Knife surgery for intractable epilepsy. J Clin Neurosci.

[7] Edmister WB, Lane JI, Gilbertson JR, Brown RD, Pollock BE (2005). Tumefactive cysts: a delayed complication following radiosurgery for cerebral arterial venous malformations. Am J Neuroradiol.

[8] Engel J, Van Ness PC, Rassmussen TB, Ojemann LM, Engel J (1993). Outcome with respect to epileptic seizures. Surgical treatment of the epilepsies.

[9] Finet P, Rooijakkers H, Godfraind C, Raftopoulos C (2010). Delayed compressive angiomatous degeneration in a case of mesial temporal lobe epilepsy treated by γ knife radiosurgery: case report. Neurosurgery.

[10] Flickinger JC, Kondziolka D, Lunsford LD, Pollock BE, Yamamoto M, Gorman DA, Schomberg PJ, Sneed P, Larson D, Smith V, McDermott MW, Miyawaki L, Chilton J, Morantz RA, Young B, Jokura H, Liščák R (1999). A multi-institutional analysis of complication outcomes after arteriovenous malformation radiosurgery. Int J Radiat Oncol Biol Phys.

[11] Foroughi M, Kemeny AA, Lehecka M, Wons J, Kajdi L, Hatfield R, Marks S (2010). Operative intervention for delayed symptomatic radionecrotic masses developing following stereotactic radiosurgery for cerebral arteriovenous malformations—case analysis and literature review. Acta Neurochir (Wien).

[12] Frazier JL, Goodwin CR, Ahn ES, Jallo GI (2009). A review on the management of epilepsy associated with hypothalamic hamartomas. Childs Nerv Syst.

[13] Ganz JC, Reda WA (2011). Radionecrosis following Gamma Knife treatment for mesial temporal lobe epilepsy. Br J Neurosurg.

[14] Georgiadis I, Kapsalaki EZ, Fountas KN (2013). Temporal lobe resective surgery for medically intractable epilepsy: a review of complications and side effects. Epilepsy Res Treat.

[15] Hensley-Judge H, Quigg M, Barbaro NM, Newman SA, Ward MM, Chang EF, Broshek DK, Lamborn KR, Laxer KD, Garcia P, Heck CN, Kondziolka D, Beach R, Salanova V, Goodman R (2013). Visual field defects after radiosurgery for mesial temporal lobe epilepsy. Epilepsia.

[16] Husain AM, Mendez M, Friedman AH (2001). Intractable epilepsy following radiosurgery for arteriovenous malformation. J Neurosurg.

[17] Kawai K, Suzuki I, Kurita H, Shin M, Arai N, Kirino T (2001). Failure of low-dose radiosurgery to control temporal lobe epilepsy. J Neurosurg.

[18] Kawamura T, Onishi H, Kohda Y, Hirose G (2012). Serious adverse effects of Gamma Knife radiosurgery for mesial temporal lobe epilepsy. Neurol Med Chir (Tokyo).

[19] Liščák R, Vladyka V, Novotný J, Brožek G, Námĕstková K, Mareš V, Herynek V, Jirák D, Hájek M, Syková E (2002). Leksell Gamma Knife lesioning of the rat hippocampus: the relationship between radiation dose and functional and structural damage. J Neurosurg.

[20] Lunsford LD, Niranjan A, Kondziolka D, Sirin S, Flickinger JC (2008). Arteriovenous malformation radiosurgery: a twenty year perspective. Clin Neurosurg.

[21] McDonald CR, Norman MA, Tecoma E, Alksne J, Iragui V (2004). Neuropsychological change following Gamma Knife surgery in patients with left temporal lobe epilepsy: a review of three cases. Epilepsy Behav.

[22] Quigg M, Harden C (2014). Minimally invasive techniques for epilepsy surgery: stereotactic radiosurgery and other technologies. J Neurosurg.

[23] Quigg M, Rolston J, Barbaro NM (2012). Radiosurgery for epilepsy: clinical experience and potential antiepileptic mechanisms. Epilepsia.

[24] Quigg M, Yen CP, Chatman M, Quigg AH, McNeill IT, Przybylowski CJ, Yan G, Sheehan JP (2012). Risks of history of diabetes mellitus, hypertension, and other factors related to radiation-induced changes following Gamma Knife surgery for cerebral arteriovenous malformations. J Neurosurg.

[25] Régis J, Peragui JC, Rey M, Samson Y, Levrier O, Porcheron D, Régis H, Sedan R (1995). First selective amygdalohippocampal radiosurgery for ‘mesial temporal lobe epilepsy’. Stereotact Funct Neurosurg.

[26] Régis J, Kerkerian-Legoff L, Rey M, Vial M, Porcheron D, Nieoullon A, Peragut JC (1996). First biochemical evidence of differential functional effects following Gamma Knife surgery. Stereotact Funct Neurosurg.

[27] Regis J, Semah F, Bryan RN, Levrier O, Rey M, Samson Y, Peragut JC (1999). Early and delayed MR and PET changes after selective temporomesial radiosurgery in mesial temporal lobe epilepsy. Am J Neuroradiol.

[28] Schröttner O, Eder HG, Unger F, Feichtinger K, Pendl G (1998). Radiosurgery in lesional epilepsy: brain tumors. Stereotact Funct Neurosurg.

[29] Schwartz TH (2010). Predicting the unpredictable: stereotactic radiosurgery and temporal lobe epilepsy. Epilepsy Curr.

[30] Shuto T, Ohtake M, Matsunaga S (2012). Proposed mechanism for cyst formation and enlargement following Gamma Knife Surgery for arteriovenous malformations. J Neurosurg.

[31] Spencer SS, Berg AT, Vickrey BG, Sperling MR, Bazil CW, Shinnar S, Langfitt JT, Walczak TS, Pacia SV, Ebrahimi N, Frobish D, Multicenter Study of Epilepsy Surgery (2003). Initial outcomes in the Multicenter Study of Epilepsy Surgery. Neurology.

[32] Srikijvilaikul T, Najm I, Foldvary-Schaefer N, Lineweaver T, Suh JH, Bingaman WE (2004). Failure of Gamma Knife radiosurgery for mesial temporal lobe epilepsy: report of five cases. Neurosurgery.

[33] Stefan H, Hummel C, Grabenbauer GG, Müller RG, Robeck S, Hofmann W, Buchfelder M (1998). Successful treatment of focal epilepsy by fractionated stereotactic radiotherapy. Eur Neurol.

[34] Talairach J, Szikla G (1965). Amygdalo-hippocampal partial destruction by yttrium-90 in the treatment of certain epilepsies of rhinencephalic manifestation. Neurochirurgie.

[35] Usami K, Kawai K, Koga T, Shin M, Kurita H, Suzuki I, Saito N (2012). Delayed complication after Gamma Knife surgery for mesial temporal lobe epilepsy. J Neurosurg.

[36] Vale FL, Bozorg AM, Schoenberg MR, Wong K, Witt TC (2012). Long-term radiosurgery effects in the treatment of temporal lobe epilepsy. J Neurosurg.

[37] Vojtěch Z, Vladyka V, Kalina M, Nešpor E, Seltenreichová K, Šemnická J, Liščák R (2009). The use of radiosurgery for the treatment of mesial temporal lobe epilepsy and long-term results. Epilepsia.

[38] Warnke PC, Berlis A, Weyerbrock A, Ostertag CB (1997). Significant reduction of seizure incidence and increase of benzodiazepine receptor density after interstitial radiosurgery in low-grade gliomas. Acta Neurochir Suppl.

