# Supplementary material for: Morphological changes after radiosurgery for mesial temporal lobe epilepsy
Source: Acta Neurochir (Wien). 2015 Aug 16;157(10):1783–92. doi: 10.1007/s00701-015-2525-2 (PMC4569650; doi:10.1007/s00701-015-2525-2)
Supplement: Supplementary file 1 — (DOCX 14 kb) [file 701_2015_2525_MOESM1_ESM.docx]

**Supplementary material Table 1:** Group’s characteristics, the presurgical evaluation protocol, and seizure outcome

| Case | SR (m/y) | Volume (mm3) | Dose (Gy) | ICH | CS | ES/39 | Surgery | ES/BO | ES/LV | FU | FU/BO | FU/AO |
| --- | --- | --- | --- | --- | --- | --- | --- | --- | --- | --- | --- | --- |
| 1 | XI/95 | 8,900 | 20 | 0 | None | IIA | None | N/A | IIIA | 217 | N/A | N/A |
| 2 | VI/96 | 7,700 | 25 | 0 | None | IIIA | XII/13 | IIIA | IA | 210 | 198 | 12 |
| 3 | XII/96 | 7,300 | 20 | ICH | p.o. (6) | IVB | II/03 | IVB | IA | 204 | 74 | 130 |
| 4 | II/97 | 6,700 | 25 | 0 | None | IVB | V/01, | IVB | IA | 178 | 51 | 127 |
| 5 | II/97 | 7,400 | 25 | 0 | None | IIIA | None | N/A | IIB | 202 | N/A | N/A |
| 6 | II/97 | 7,600 | 25 | ICH | i.v. (3) | IIIA | X/13 | IVB | IA | 202 | 188 | 14 |
| 7 | IX/97 | 7,600 | 25 | ICH | i.v. (3) | IIIA | VI/06 | IVB | ID | 195 | 93 | 102 |
| 8 | XI/97 | 6,600 | 25 | 0 | None | IVC | III/01 | IIIA | IA | 193 | 40 | 153 |
| 9 | IV/98 | 5,200 | 18 | 0 | None | IVB | VI/07 | IVB | IIC | 188 | 98 | 90 |
| 10 | XI/98 | 5,900 | 18 | 0 | None | IVB | None | N/A | IVB | 181 | N/A | N/A |
| 11 | XI/98 | 5,700 | 18 | 0 | None | IVB | None | N/A | IVB | 181 | N/A | N/A |
| 12 | XII/98 | 6,000 | 18 | 0 | None | IIIA | XI/05 | IVC | IA | 180 | 83 | 97 |
| 13 | V/99 | 6,100 | 18 | 0 | None | IIIA | VI/04 | IVA | IIC | 175 | 61 | 114 |
| 14 | V/99 | 6,000 | 18 | 0 | None | IIIA | None | N/A | IB | 175 | N/A | N/A |

**Abbreviations:** CS, corticostereoid treatment (duration in months in the bracket); ES/39, Engel Class at 39 months after GKRS; ES/BO, Engel Class before operation; ES/LV, Engel Class at last visit; FU, total length of follow-up in months; FU/AO, length of follow-up after operation in months; FU/BO, length of follow-up before operation in months; ICH, signs of intracranial hypertension; i.v., intravenously; N/A, not applicable; p.o., perorally; SR (m/y), month/year of GKRS; surgery, month/year of open surgery;

**Note:** Patient 4 died in XII/12 from influenza.
